# Molecular Engineering of MXene-Covalent-Triazine Framework Interfaces for Electrochemical Actuators

**DOI:** 10.1021/acsnano.5c04154

**Published:** 2025-07-01

**Authors:** Manmatha Mahato, Sanghee Nam, Geetha Valurouthu, Hyunjoon Yoo, Mousumi Garai, Ji-Seok Kim, Woong Oh, Jawon Ha, Vipin Kumar, Chi Won Ahn, Yury Gogotsi, Il-Kwon Oh

**Affiliations:** † National Creative Research Initiative for Functionally Antagonistic Nano-Engineering, Department of Mechanical Engineering, 34968 Korea Advanced Institute of Science and Technology (KAIST), 291 Daehak-ro, Yuseong-gu, Daejeon 34141, Republic of Korea; ‡ Department of Materials Science & Engineering, and A. J. Drexel Nanomaterials Institute, 6527 Drexel University, Philadelphia, Pennsylvania 19104, United States; § National Nanofab Center (NNFC), Korea Advanced Institute of Science and Technology (KAIST), 291 Daehak-ro, Yuseong-gu, Daejeon 34141, Republic of Korea

**Keywords:** MXene, CTF, functional active materials, supercapacitors, actuators

## Abstract

Developing multifunctional nanomaterials for soft electrochemical actuators and energy storage devices is crucial for advancing next-generation soft robotics, wearable electronics, and bioinspired technologies. However, existing electrode materials face fundamental trade-offs among electronic conductivity, charge storage capacity, and ion transport efficiency. Here, we report a molecularly engineered hybrid nanoarchitecture that achieves the physicochemical stabilization of MXene terminals by the *in situ* growth of 4*H*-pyran functionalized, electronically conjugated covalent-triazine frameworks (MXene-CTF). The integration of MXene and CTFs forms a synergistic active electrode for superior supercapacitors and actuators by offering significantly enlarged interactive surface areas, a well-developed network of nanoporous channels, and enhanced electrical conductivity. The MXene-CTF electrode provides an eminent energy density of 159.8 Wh kg^–1^ at a power density of 150 W kg^–1^ in a supercapacitor configuration with a nonaqueous ionic liquid electrolyte. Also, it achieves a bending strain of 1.1% and a blocking force of 5.8 mN, with a rapid response time of 1.4 s and a phase delay of 0.15 rad under an ultralow input potential of 0.5 V in a soft actuator configuration. This work unveils a strategy for the molecular-level synergistic integration of MXene with CTFs, offering a promising pathway for the development of high-performance energy storage and electrochemical actuation technologies.

## Graphical abstract

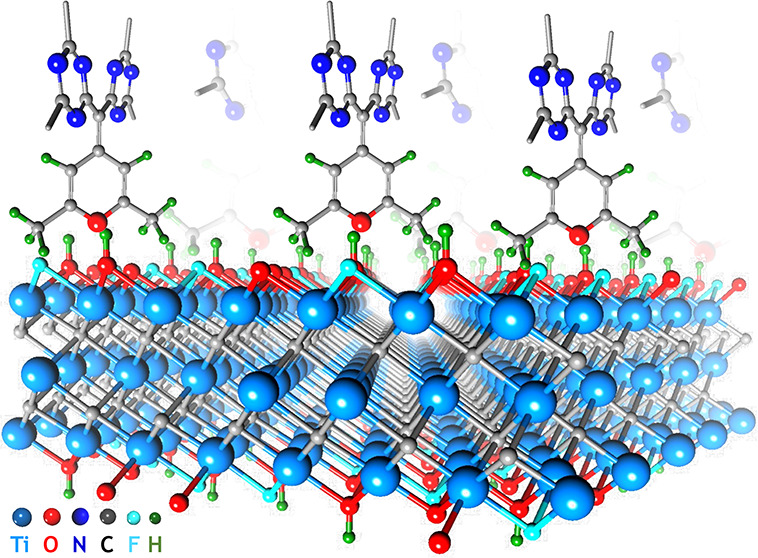

## Introduction

The development of next-generation electric vehicles,^1–3^ flexible electronics,^4–7^ active biomedical devices,^8–10^ and adaptive wearable technologies^11–13^ relies on the advancements in electrochemical energy storage devices and electromechanical actuators. Despite significant advancements in capacitors and actuators, designing multifunctional active electrode materials that simultaneously fulfill multiple performance criteria in electrochemical devices remains a critical challenge. Consequently, many innovations in heteronanostructured materials remain largely theoretical and have yet to be translated into practical applications. This limitation arises primarily from the inherent difficulties in developing hybrid electrode architectures that integrate essential properties and often exhibit conflicting material requirements. For example, high-performance electrochemical devices require active electrode materials with robust stability under electrochemical conditions and exceptionally high electrical conductivity and ionic capacitance. However, in practice, materials that offer metallic-level electrical conductivity typically exhibit lower electrochemical ionic capacitance, and conversely, materials with high ionic capacitance usually possess limited electrical conductivity.

Recent advancements in MXene and carbon electrode materials for high-performance electrochemical devices offer a promising prospect for MXene-based electronics (MXetronics)^14,15^ and carbon-enabled electronics,^16–18^ respectively. MXene, especially Ti_3_C_2_T_*x*_, possesses metal-like electrical conductivity and a two-dimensional (2*D*) interactive surface, yet their practical deployment is hindered by rapid oxidation and limited surface stability in electrochemical environments.^19–23^ Although efforts have been made to stabilize MXene surface terminations through electronic interactions with discrete organic functional molecules, these approaches have not significantly enhanced electrochemical capacitive properties.^24^ In some cases, they even led to performance degradation over time under ambient conditions.^24–26^ Meanwhile, advancements in carbon-architectured materials and electronically conjugated covalent-triazine frameworks (CTFs) exhibit long-term stability and high ionic specific capacitance, but suffer from relatively poor electrical conductivity.^27–33^ Conventional strategies, such as physical mixing of MXenes with CTFs, fail to provide long-term stability and synergistic charge storage in electrochemical devices. This situation necessitates precise nanoengineering of the active hybrid electrode materials to balance all the necessary properties, such as stability, electrical conductivity, and ionic capacitance, crucial in electrochemical supercapacitors and actuators.

In recent years, numerous attempts have been made to improve the electrochemical stability of MXenes, which consist of 2D transition metal carbides, nitrides, or carbonitrides, for their potential applications.^34,35^ For example, a series of stable 2D metal-intercalated layered carbides have been proposed by removing the MXene terminals using a chemical scissor method.^36^ The O–B–O termination of MXene has been found to have higher stability and ionic charge storage capacity for electrochemical devices.^37^ The amido- and imido-terminated Ti_3_C_2_T_*x*_ MXene has also exhibited higher stability against oxidation.^38^ Similarly, CTF with surface nanoengineering by confined ion channels has shown substantial improvements in both the electrical conductivity and electrochemical energy efficiency.^39^ CTFs have been extensively configured in recent years by optimizing surface functionalities and backbone structures to improve the overall electrochemical performance.^40–42^ In our previous report, 4*H*-pyran units in resonance with the triazine frameworks of a CTF were shown to significantly enhance and outperform the ionic charge storage capacity in a solid-state flexible supercapacitor configuration.^43^ Progress in the structural optimization of CTFs, which include electronic conjugation and surface heteroatoms (N and O), shows substantial advancement in supercapacitors and actuators.^43–45^ It is noteworthy that both MXene and CTF are excellent choices for electrochemical devices.

However, although conventional MXenes demonstrate high electrical conductivity, they suffer from limited ion storage capacity and electrochemical stability. In contrast, CTFs provide high storage capacity, and their electronically conjugated structures enhance electrical conductivity to some degree but not to the extent required for superior performances. These issues must be addressed promptly to ensure their practical applicability. Here, we uncover a fundamentally alternative strategy for synergistic stabilization of MXene surfaces via an *in situ* grown 4*H*-pyran functionalized CTF, forming an electronically and physically conjugated hybrid interface. Fully delaminated 2D layers of Ti_3_C_2_T_*x*_ MXene are stabilized by 4*H*-pyran functional moieties via the *in situ* growth of a π-conjugated CTF through a degassed ionothermal process. This molecular-level synergistic interaction enhances ion transport and electrical conductivity, and prevents oxidation of MXenes, yielding a system with record electrochemical stability and actuator performance. This molecular-level integration of MXene and CTF effectively addresses the distinct challenges in electrochemical devices, opening alternative avenues for developing the MXene–CTF hybrid electrode with diverse structural configurations.

## Results and Discussion

### Stabilization of Ti_3_C_2_T_*x*_ MXene by 4*H*-Pyran-Functionalized CTF at the Molecular Level

Prior to undertaking the electronic stabilization process via *in situ* growth CTF, the delaminated Ti_3_C_2_T_*x*_ MXene ink was freeze-dried to maintain the structural integrity.^46^ It was then transferred to an argon-filled glovebox and placed in a glass ampule with 4-(dicyanomethylene)-2,6-dimethyl-4*H*-pyran and anhydrous zinc chloride. The charged ampule was degassed completely and flame-sealed under vacuum before the ionothermal growth of the 4*H*-pyran functional CTF, as shown in Figure 1a. A comprehensive discussion of the experimental parameters and their roles is provided in the Methods section. The hydroxyl terminations of each delaminated 2D layer of MXene establish electronic communication via hydrogen bonding (H–bonding), which induces the 4*H*-pyran units to undergo a sequential growth of the CTF as schematically represented in Figure 1a. This unique synergy of MXene and CTF produces a robust electroactive material, MXene–CTF, with high electrochemical stability, improved ion storage capacity, and ultrafast electro-ionic conduction. The significant enhancement of multiple key electrochemical properties within a single active electrode material facilitates the development of supercapacitors and actuators with optimized energy densities and superior actuation performance. Figure 1b schematically illustrates the suitability of integrating MXene and CTF for both the high-level conduction of electrical charge and the transport of ions during the electrical switching process. As illustrated by plausible chemical drawings, the MXene can accelerate the rate of ion conduction due to its metal-like electrically conductive path. The CTF counterpart of MXene–CTF not only enhances ion storage capabilities but also electronically stabilizes the MXene terminations. This is achieved by introducing nanoporous structures and 4*H*-pyran functional surfaces, which together optimize the material’s performance. The successful growth of CTF on the 2*D*-layer surfaces of MXene is evident in the ultrahigh resolution transmission electron microscopic (TEM) images at the few-nanometer scale (Figure 2a–f). The nanoscale TEM images of MXene–CTF demonstrate the presence of 4*H*-pyran CTF, in addition to Ti_3_C_2_T_*x*_ MXene, by reflecting the dominant amorphous structural characteristics of CTF in comparison with the crystalline nature of MXene. Figure S1a,d displays TEM images of the MXene–CTF active materials at lower magnifications. The images display that CTF has a robust affinity for MXene terminations, which can provide comprehensive electronic stability against oxidation. Furthermore, the mesoporous-dominant surfaces of CTF facilitate stress-free ion movement in electrochemical soft actuators under electrochemical switching. To further ensure the structural identity of MXene–CTF at the atomic level appearances, high-angle annular dark-field (HAADF) scanning TEM with mapping of building-block elements was constructed at this nanoscale configuration (Figure 2g–l). It was observed that the mapping images for the backbone elements of MXene-CTF, namely, carbon (Figure 2h), oxygen (Figure 2i), nitrogen (Figure 2j), titanium (Figure 2k), and fluorine (Figure 2l), precisely align with the nanoscale HAADF tomography image of it (Figure 2g). In addition to stability, this molecular-level integration of CTF significantly expanded the electrolyte-accessible active surface area of MXene–CTF, thereby establishing an advanced platform for the development of supercapacitors and actuators. The specific surface area of MXene–CTF active electrode material was determined to be as high as 1224 m^2^ g^–1^ by the conventional Brunauer–Emmett–Teller (BET) theory (Figure 3a), which was more than 44 times that of pristine Ti_3_C_2_T_*x*_ MXene (27.6 m^2^ g^–1^). The argon physisorption isotherm with its distinctive hysteresis loop at relative pressures (*p*/*p*_0_) ranging from 0.4 to 0.8 provides evidence of electrochemically favorable mesoporous–dominant surfaces, along with hierarchical porosity. The limited accessibility of electrolyte ions in pristine MXene, characterized by its predominantly microporous structural surface with a pore diameter of 1.48 nm (Figure S2), is completely overcome in the MXene–CTF configuration. The integration of CTF onto the MXene surface resulted in the formation of an advanced electrode material with highly electroactive surfaces and dominant mesoporous structures, with pore diameters ranging from 2.0 to 5.0 nm, which were accompanied by micropores (Figure 3b). It is postulated that this novel nanostructured and enlarged active surface of MXene–CTF has the ample potential to fulfill all the necessary parameters as required for supercapacitors and actuators. However, prior to electrochemical applications, it is equally important to understand and elucidate the electronic interaction between the surface functionals of MXene and CTF. The fundamental realization of the functional sites allows for the identification of the possible electronic interactions, which are limited to H–bonding only. This interaction is enabled by the heteroatoms, oxygen, and nitrogen from 4*H*-pyran CTF, which utilize their nonbonding electrons to interact with the electron–deficient hydrogen of the surface hydroxyl of Ti_3_C_2_T_*x*_ MXene. Consequently, to ascertain the alterations in the electronic state of oxygen and nitrogen in MXene–CTF relative to their pristine states, X-ray photoelectron spectroscopy (XPS) analysis was conducted (Figures 3c,d and S3). The O 1s XPS spectra indicated that the oxygens from 4*H*-pyran units of CTF were substantially involved in the H–bonding electronic interaction with the hydroxyl proton of MXene. As the binding energy of pristine CTF for O 1s was shown to increase from 531.92 to 532.59 eV, while that of MXene was reduced from 531.32 to 529.97 eV in MXene–CTF (Figure 3c). In contrast, it was observed that there were minimal alterations in the binding energies for the nitrogen configurations in both pristine CTF and MXene–CTF (Figure 3d). The three typical nitrogen configurations, namely, N_graphitic_ (in violet), N_cyano_ (in blue), and N_triazine_ (in olive), exhibited comparable XPS signals in both materials. This observation corroborates the hypothesis that the oxygens from 4*H*-pyran functionalized CTF were solely engaged in the electronic interactions with the MXene, while the nitrogen remained uninvolved due to steric constraints (Figure 1a). FTIR and Raman spectroscopic analyses further substantiate the hypothesis of interfacial interactions, as detailed in the Supporting Information (Figure S4a,b). It is plausible that the electron-rich accessible nitrogen centers of MXene–CTF contributed considerably to the overall active surface charge, thereby enhancing electrochemical ion storage (Figure 1a).

**Figure 1:**
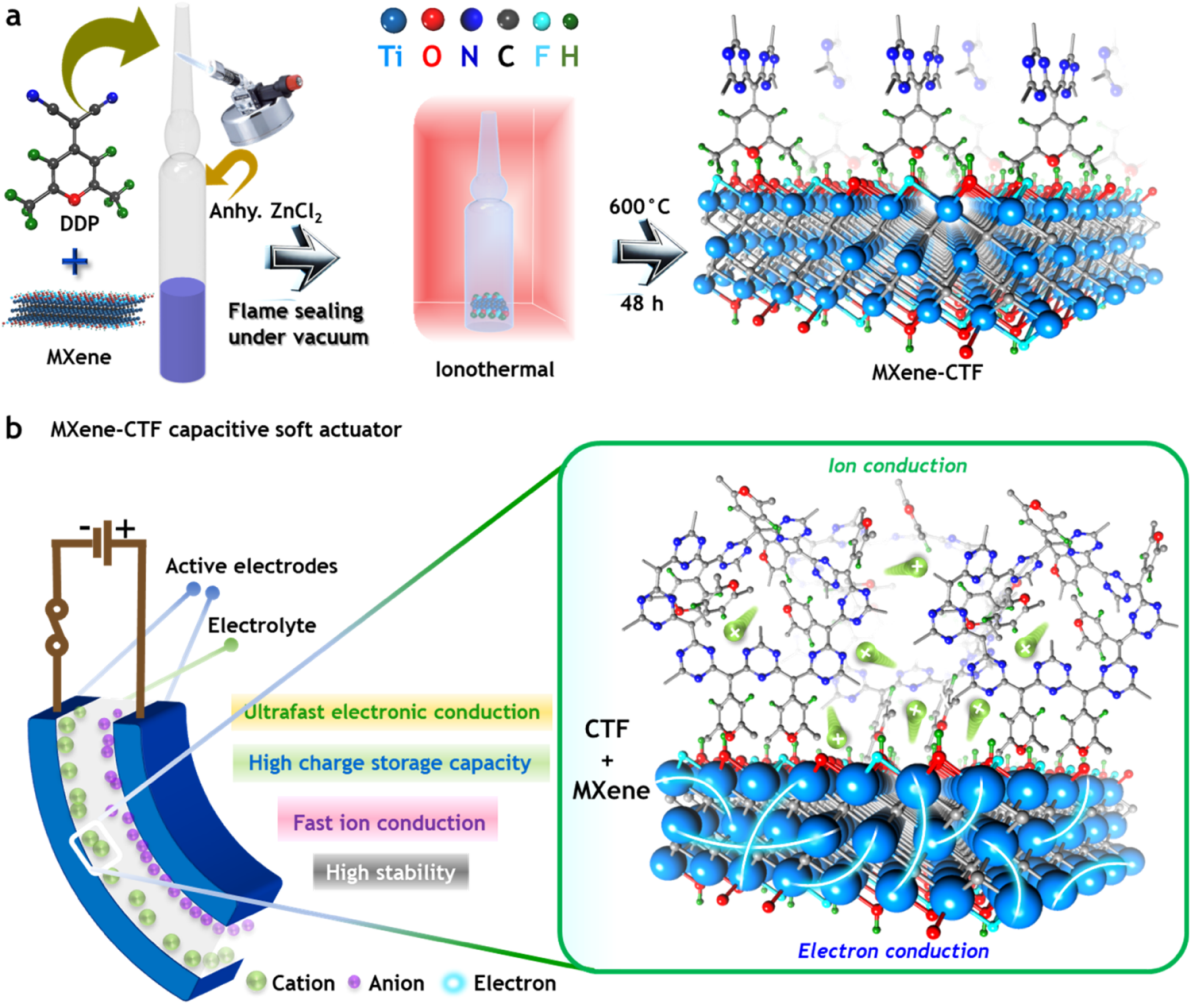
Schematic illustration for MXene-CTF with plausible electronic interaction and importance for developing electrochemical capacitive actuator. (a) In-depth experimental parameters for synthesizing MXene-CTF active material and possible interacted chemical structure of it. (b) Sketch to highlight the functional importance of MXene-CTF active electrode material in order to improve the electrochemical ions conduction for both the supercapacitor and soft actuator configurations.

**Figure 2:**
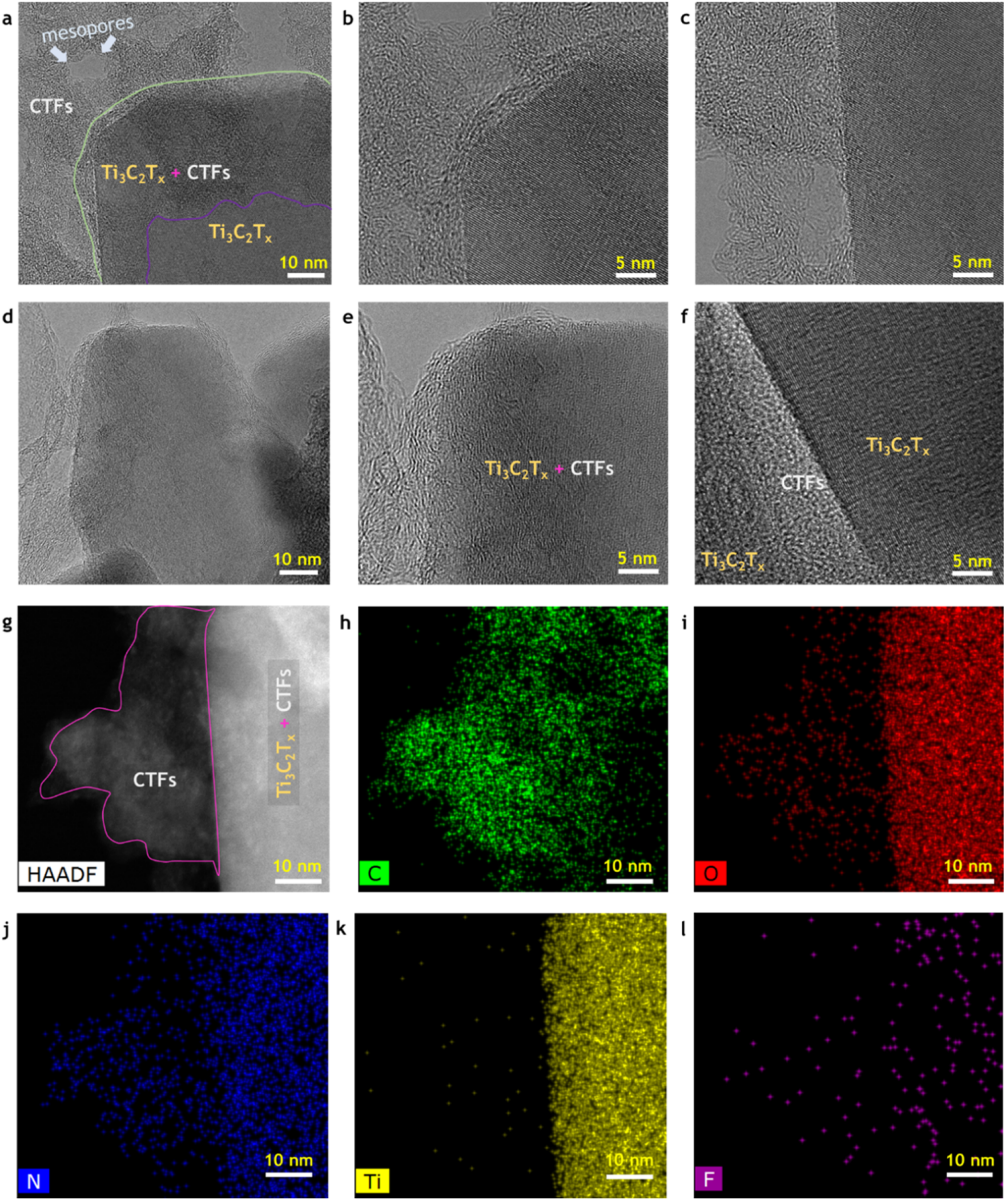
Few-nanometer-scale structural characterization of MXene-CTF active electrode materials. (a–f) TEM images of MXene-CTF at nanoscale, displaying the growth of 4*H*-pyran functional CTF onto the 2D layer of MXene surface. (g–l) STEM tomography with corresponding elemental mapping images of the MXene-CTF, displaying the molecular scale integration of MXene and CTF. The structural elements were orderly according to the tomography image.

**Figure 3:**
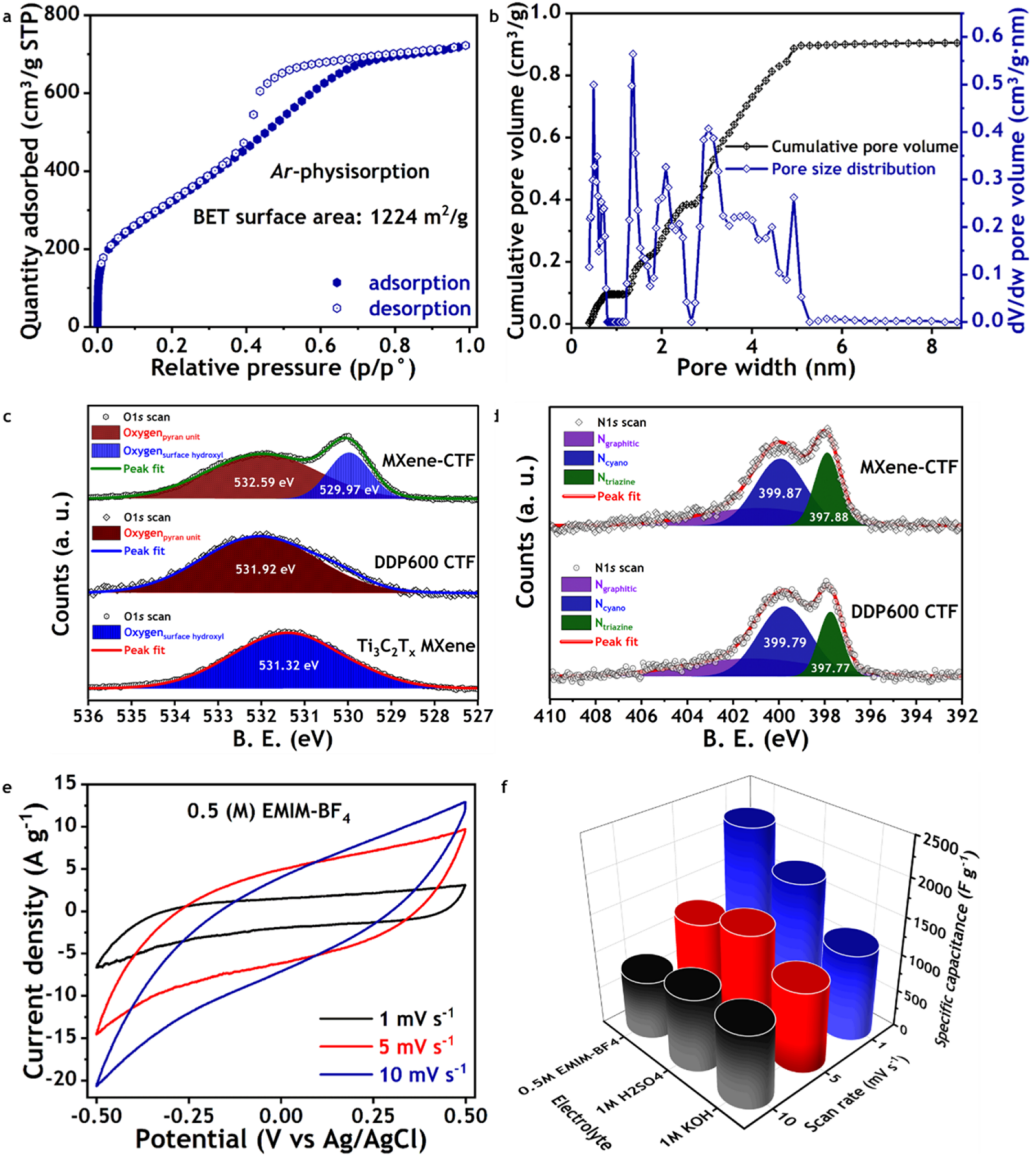
Physicochemical and electrochemical characterization of MXene-CTF active electrode material. (a, b) Ar-physisorption and pore-size distribution analyses of MXene-CTF. Display hierarchical porosity. (c, d) Comparative XPS analyses of MXene, CTF, and MXene-CTF to understand the electronic interaction between MXene and CTF in their atomic configurations. Display substantial electronic interaction between the 4*H*-pyran units and surface hydroxyl of MXene. (e, f) Electrochemical charge storage–discharge profiles and capacity analyses at both the aqueous and nonaqueous electrolyte media. Display dominant EDLC properties with significantly increased specific capacitance.

### Evolution of Electrochemical Capacitive Properties of MXene–CTF Active Material

The performance of supercapacitors and actuators primarily depends on the active electrode materials’ electrochemical charge storage and discharge characteristics. Cyclic voltammetry (CV) measurement is one of the best tools to understand the electrochemical capacitive properties of an active material. To determine the electrolyte-specific electro-ionic efficiency of the novel MXene–CTF active material, CV measurements were performed in aqueous KOH and H_2_SO_4_ (1.0 M), as well as in organic EMIM-BF_4_/acetonitrile (0.5 M) solution at different scan rates (Figures S5a,b and 3e). Mesoporous-dominant hierarchical nanoporous surfaces with electron-rich nitrogen centers of MXene–CTF resulted in typical electrochemical CV responses with the combination of electrical double-layer capacitance (EDLC) and pseudocapacitance. As anticipated, the current density was markedly elevated in a three-electrode configuration, with Ag/AgCl and platinum mesh serving as the reference and counter electrodes, respectively. At a scan rate of 10 mV s^–1^ under a constant electrochemical potential window from −0.5 to +0.5 V, the MXene–CTF exhibits an instrumental ionic specific capacitance of 687, 674, and 626 F g^–1^ in aqueous KOH, H_2_SO_4_, and organic EMIM-BF_4_/acetonitrile electrolyte, respectively (Figure 3f). It is noteworthy to mention that the ionic capacitances of pristine MXene and CTF, respectively, were 271 and 166 F g^–1^, under the same electrochemical environment in the EMIM-BF_4_/acetonitrile electrolyte solution (Table S1). The specific capacitance values directly reflect the enhancement of the electrochemical capacitive properties of MXene–CTF as a result of the integration of 4*H*-pyran functionalized CTF on the 2D-layer surfaces of MXene, compared to their individual configurations. Table S2 presents a comparative analysis of the specific capacitance values of high-performance active electrode materials in nonaqueous electrolytes, wherein MXene–CTF has been identified as the leading material to date.^6,25,26,43–45,47a–47h^ The exceptional capacitive properties of MXene–CTF have prompted us to explore its potential for use in supercapacitors and actuators with the aim of elucidating its practical utility.

### Electrochemical Supercapacitor Performance of MXene–CTF Active Material

As the demand for efficient, durable, and rapid-charging energy storage solutions continues to grow, supercapacitors are poised to play a pivotal role in the next generation of portable devices. Their distinctive properties complement traditional batteries, fostering the development of hybrid systems that enhance the performance and reliability in consumer electronics and wearables. It is important to highlight that the two primary ion storage principles driving supercapacitor technology are EDLC and pseudocapacitance. In EDLC, the electrolyte ions or charged molecules are intercalated or deintercalated at the electrode/electrolyte interface during the switching of an electrical signal, resulting in a moderate ion-specific capacitance with exceptional cycle life. In contrast, pseudocapacitance involves a faradic charge transfer mechanism via reversible redox reactions, which results in a very high specific capacitance. However, the storage capacity is diminished during the progression of continuous charging and discharging cycles due to the spontaneous loss of electronic charges in each redox cycle. Consequently, supercapacitors based on pseudocapacitance exhibit a relatively lower power density and cycle life. Therefore, researchers persistently strive to identify an active electrode material that exhibits both high ionic capacitance and long cycle life, with the ultimate goal of further enhancing supercapacitive performance. Integrating 4*H*-pyran functionalized CTF into the MXene surface synergistically combines EDLC and pseudocapacitance. This dual-capacitive mechanism enhances the electro-ionic capacitance and prolongs the cycle life by electronically stabilizing the active surface terminations of MXene. Furthermore, the employment of an EMIM-BF_4_ ionic liquid as an electrolyte in the MXene–CTF supercapacitor markedly expands the operational potential window compared to traditional aqueous electrolytes, thereby maximizing the energy and power densities. The use of ionic liquids allows the potential window to be widened to a maximum of 3.0 V, extending from −0.5 to 2.5 V compared to the narrow potential window of 1.0 V observed for aqueous electrolytes. The electrochemical CV response patterns in EMIM-BF_4_ electrolyte under a wider range of scan rates, starting from very low (0.5 mV s^–1^) to extremely high (1000 mV s^–1^), provide evidence in support of the combined EDLC and pseudocapacitive ion storage mechanism of MXene–CTF symmetric supercapacitor (Figures 4a and S6a,b). The quasi-rectangular CV responses observed at lower scan rates (0.5–50 mV s^–1^) and spindle-like shapes at higher scan rates (75–1000 mV s^–1^) confirm the active involvement of 4*H*-pyran functionalized CTF and electronically stabilized MXene surfaces with the electrolyte ions. The output CV responses of the MXene–CTF supercapacitor are consistent with the hypothesis that minimal faradaic charge transfer occurs during electrochemical ion storage or discharge cycles. These findings support the electronic stability of MXene surface terminals and indicate the potential for enhanced long-term electrochemical capacity retention. The galvanostatic charge–discharge (GCD) profiles of the MXene–CTF supercapacitor were subjected to meticulous analysis to ascertain the energy storage capacity in terms of specific capacitance across a comprehensive range of constant current densities, commencing from 0.1 to 5.0 A g^–1^ (Figures 4b and S7a,b). Figure 4b illustrates the typical triangular-shaped GCD profile up to a constant input current density of 0.75 A g^–1^, exhibiting a substantially high ionic specific capacitance of 127.8 F g^–1^ (≈570 F m^–2^) at 0.1 A g^–1^ (loaded mass of active MXene–CTF: 2.4 mg cm^–2^). The specific capacitance values calculated by analyzing the discharge profiles of MXene–CTF supercapacitor at different input current densities after the IR drops are tabulated in Table S3. Notwithstanding the universal tendency for specific capacitance values to decline with increasing input current densities, the MXene–CTF supercapacitor exhibited the ability to maintain capacitance above 100 F g^–1^ even when subjected to relatively high input current densities of 1.0 A g^–1^ (Table S3). When evaluated against the performance of bare 4*H*-pyran functionalized CTF and Ti_3_C_2_T_*x*_ MXene-only supercapacitors, the specific capacitance and its retention exhibited consistent and substantial improvement in MXene–CTF (Figure 4c). This improvement in the electrochemical ion storage capacity for the MXene–CTF supercapacitor was also evident in the comparative output energy densities against the bare CTF and MXene (Figure 4d). The MXene–CTF exhibited an energy density of 159.8 Wh kg^–1^ at a power density of 150 W kg^–1^, demonstrating superior supercapacitive performance compared to both the CTF and MXene counterparts (Figure 4d). The enhanced diffusion rate of electrolyte ions at the electrode–electrolyte interfaces can be attributed to the synergistic coupling of the mesoporous-dominant large active surfaces of CTF and the suitable electron conduction paths of MXene (Figure 4e). As illustrated in the electrochemical impedance spectra (EIS), the diffusivity of electrolyte ions is highest for the MXene–CTF supercapacitor, particularly in the lower-frequency region, despite the marginal increase in charge transfer resistance from bare MXene (Figure 4e). The surface contact resistances were found to be nearly identical for bare MXene and MXene–CTF, while exhibiting a slight increase for bare CTF, which were 4.0, 4.3, and 5.3 Ω, respectively. The Ragone plot demonstrated that the proposed MXene–CTF exhibited the highest supercapacitive performance in terms of both specific energy and power density when compared to recently developed high-performance supercapacitors that employ ionic liquid as a source of active electrolyte ions (Figure 4f).^43,48^ In terms of electrochemical stability, the MXene–CTF supercapacitor exhibited an exceptional capacity for ion storage, demonstrating notable retention over extended periods. At a constant input current density of 1.0 A g^–1^, the supercapacitor exhibited a cyclic retention of 96.4% after 10,000 continuous charge–discharge cycles (Figure 4g). As illustrated in the inset of Figure 4g, the GCD profiles confirm the presence of identical electrochemical response patterns during the initial cycle, the 2000th cycle, the 4000th cycle, the 6000th cycle, and the 10,000th cycle. Given the ultralong stability of charge–discharge cycles exhibited by the MXene–CTF supercapacitor, we sought to ascertain its potential for application in electrochemical soft actuation within the same electrolyte embedded into a Nafion membrane, to gauge its practical importance.

**Figure 4:**
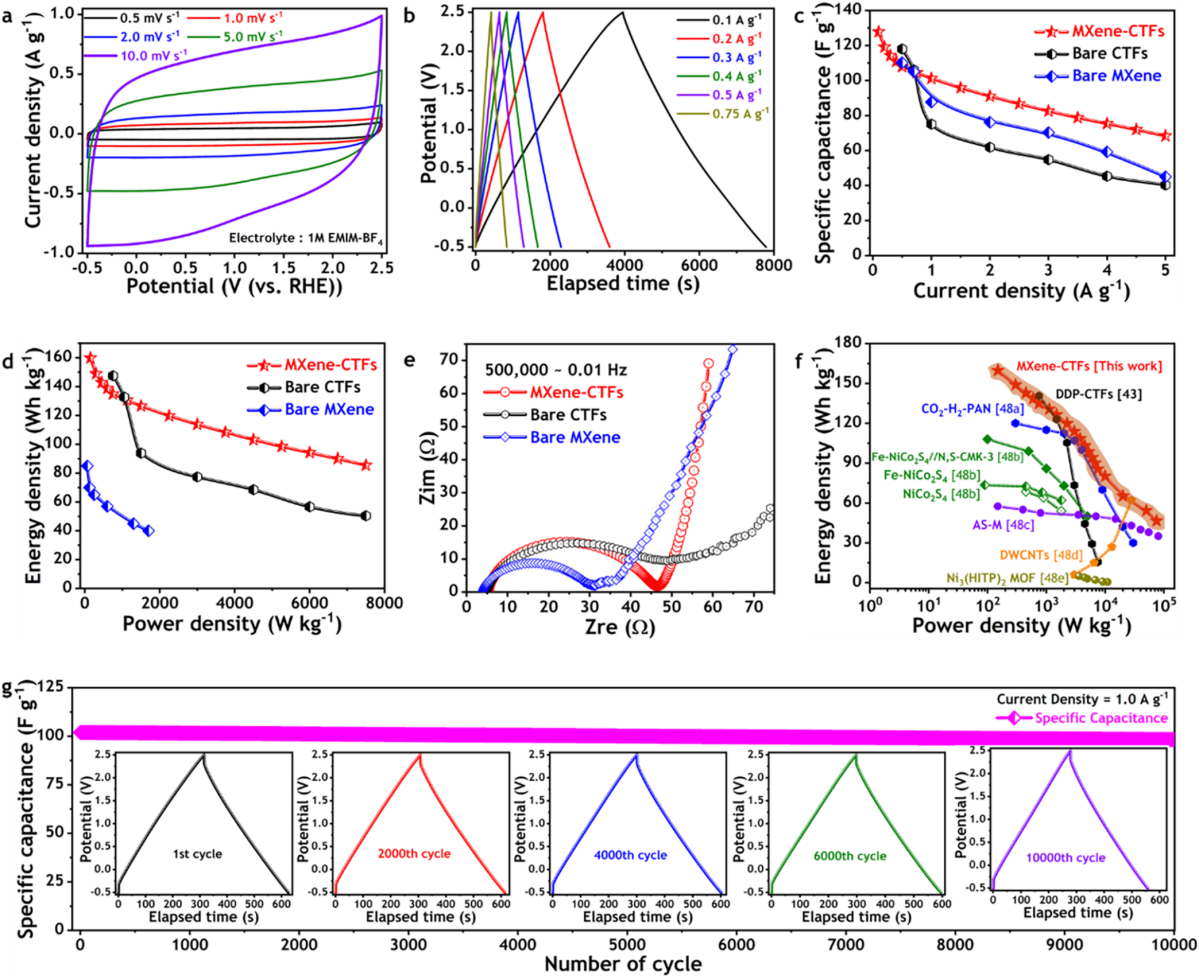
Electrochemical capacitive performances of MXene-CTF-based supercapacitor in nonaqueous electrolyte in comparison with its counterparts and up-to-date analogous available literature. (a) Cyclic voltammetry analysis of MXene-CTF supercapacitor in an EMIM-BF_4_/acetonitrile electrolyte solution. The scan rates range from 0.5 to 10 mV s^–1^, with a fixed potential window of −0.5 to 2.5 V. (b) Galvanostatic charge–discharge response profiles of MXene-CTF supercapacitor at different current densities, with the wider potential window of −0.5 to 2.5 V. (c) Comparison of the ionic specific capacitance for the MXene-CTF supercapacitor with its parent materials, MXene and CTF, displaying substantially larger performances values and retention with increasing input current densities. (d) Comparative Ragone plots of MXene, CTF, and MXene-CTF supercapacitors, displaying the performance improvements of MXene-CTF compared to its individual components. (e) Comparison of electrochemical impedance spectra for MXene, CTF, and MXene-CTF supercapacitors. (f) Comparative performance analysis of MXene-CTF supercapacitor with other analogous materials, focusing on both the values of energy density and power density (Ragone plot). (g) Long-cycle retention of specific capacitance for the MXene-CTF supercapacitor up to 10,000 cycles at a constant current density of 1.0 A g^–1^, displaying the ultrastability and durability of the supercapacitor over extended use.

### Electrochemical Actuation Performance of MXene–CTF Active Material

The advancement of electrochemical capacitive properties in MXene–CTF has substantially impacted the pivotal actuation characteristics of a soft actuator, rendering it more practical for robotics applications (Figure 5a–i). Actuators are the integral components of soft robots and must exhibit robust stability in performance, durability under working conditions, and suitable mechanical properties during actuation, including strain, force, and controlled rapid displacement with minimal phase delays. To gain insight into the mechanical deflection in terms of displacement under ultralow input potential (≤0.5 V), the MXene–CTF-based soft actuator (MXene-CTF-PP) was excited at a constant frequency of 0.1 Hz with varied voltages, starting from 0.01 to 0.5 V (Figure 5a). In open-air, a controlled mechanical deflection was observed with the increase in applied voltages, reaching a peak-to-peak bending displacement of 17.3 mm at 0.5 V, while showing substantial actuation responses were evident even at 0.03 V. To ensure a valid comparison and determine the precise impact of MXene–CTF to actuation performance, an identical PEDOT-PSS-only soft actuator (PP) was constructed (discussion on the experimental fabrication processes and their dimensions is provided in the Methods section). The MXene-CTF-PP soft actuator exhibited a peak-to-peak bending displacement three times (300%) that of PP when excited at 0.1 Hz frequency with a ± 0.5 V square wave input potential (Figure 5b). The incorporation of MXene–CTF into the MXene-CTF-PP soft actuator resulted in two significant advancements when stimulated under a 0.5 V direct current (DC). First, the mechanical bending of PP increased from 3.6 to 10.7 mm due to the expansion of effective electrolyte surfaces, which is approximately 3-fold. Second, actuation speed was enhanced up to more than 5-fold due to the MXene-induced electronic conduction, where the original response time of PP soft actuator was reduced from 7.2 to 1.4 s in MXene-CTF-PP (Figure 5c). Optical images of the actuation setup and the MXene-CTF-PP soft actuator operating under a DC voltage of +0.5 V are presented in Figure S8a,b. Furthermore, Movie S1 visually demonstrates the mechanical deflections of the actuator under various applied voltages at a fixed excitation frequency of 1.0 Hz.

**Figure 5:**
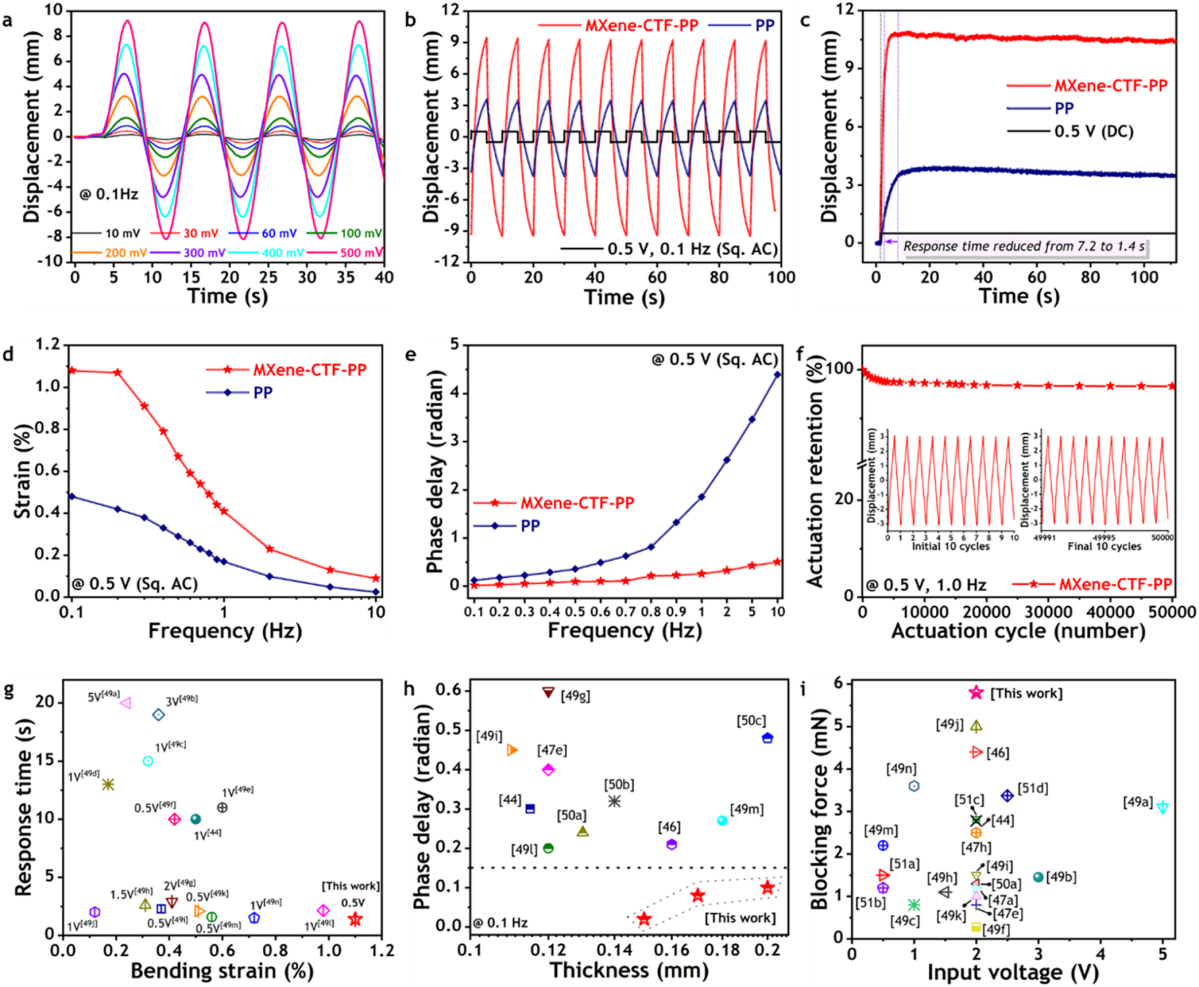
Electrochemical actuation performances of MXene-CTF-based soft actuator (MXene-CTF-PP) in nonaqueous electrolyte under open environment in comparison with its counterpart and up-to-date available literature. (a) Time-dependent mechanical bending of the MXene-CTF-PP soft actuator under incremental ultralow input voltages ranging from 0.01 to 0.5 V at a constant excitation frequency of 0.1 Hz. (b) Characteristic improvement of bending responses for MXene-CTF-PP compared to the analogous PP-only soft actuator when excited at 0.5 V and 0.1 Hz. (c) Comparison by showing advancement in actuation speed and deflection for MXene-CTF-PP with the analogous PP soft actuator at 0.5 V ultralow DC input potential. (d) Bending strain improvement of MXene-CTF-PP compared to the analogous PP soft actuator at 0.5 V square AC under a wide range of excitation frequencies, from 0.1 to 10 Hz. (e) Reduction of phase delay during actuation for MXene-CTF-PP soft actuator compared to the PP at 0.5 V square AC under a wide range of excitation frequencies, from 0.1 to 10 Hz. (f) Long-cycle retention of actuation performance for the MXene-CTF-PP soft actuator up to 50000 cycles under continuous excitation at 0.5 V and 1.0 Hz. The inset displays the initial and final ten cycles of bending responses. (g–i) Overall electroactive actuation performance advancements of MXene-CTF-PP compared to current high-performance soft actuators, considering key parameters such as (g) response time and bending strain, (h) phase delay and thickness, and (i) blocking force and input voltage.

In light of the difficulties encountered in developing ultralow-voltage-driven electro-ionic soft actuators, it is worth noting that the necessary bending strain for practical applications remains unattainable. This is mainly due to the inherent limitations of the actuation speed and stiffness in the active actuators. The intriguing actuation properties with a fast-rising speed of MXene-CTF-PP enable the achievement of an instantaneous bending strain difference of 1.1% at only 0.5 V, which is nearly 2.3-fold that of the PP soft actuator (Figure 5d). As illustrated in Figure 5d, the incorporation of MXene–CTF active material markedly enhances the mechanical strain in comparison to the similar PP soft actuator across a broad range of excitation frequencies (0.1–10.0 Hz). Another crucial aspect of an ideal electro-ionic soft actuator is the necessity for minimal phase delays during actuation under alternative currents. This will facilitate the effective control of soft actuators when a synchronized design process is implemented for real-world robotic applications. It was observed that the phase delay of the MXene-CTF-PP soft actuator was significantly reduced, with almost negligible delay up to 0.7 Hz. Thereafter, it was considerably lower compared to PP when excited at a 0.5 V square wave input potential up to a maximum of 10.0 Hz (Figure 5e). The phase delay can be defined as the time lag between the signal of the input voltage and the output mechanical actuation responses. This lag is directly proportional to the rate of ionic charge storage and discharge capacity during voltage switching. The MXene–CTF exhibits well-defined nanoporous active surfaces with electronically conductive paths, which facilitate the rapid accommodation and transportation of electrolyte ions during the switching of electrical signals. The robust stability of MXene–CTF is attributed to the strong electronic interaction between the Ti_3_C_2_T_*x*_ MXene surface and the 4*H*-pyran functionalized CTF. This interaction has demonstrated significant potential for achieving ultradurable actuation in open-air environments. The MXene-CTF-PP soft actuator exhibited a remarkable actuation retention of 97.0% even after undergoing 50,000 continuous charge–discharge cycles at an input potential of 0.5 V and an excitation frequency of 1.0 Hz (Figure 5f). Most notably, it demonstrated the ability to deliver a peak-to-peak bending displacement of 6.0 mm at this ultralow voltage and relatively high excitation frequency (inset of Figure 5f). In contrast, the Ti_3_C_2_T_*x*_ MXene-based soft actuator has demonstrated a consistent decline in actuation performance, with a previously reported actuation retention of only 86.0% after 10,000 cycles.^46^ The high-resolution cross-sectional SEM images of the MXene-CTF-PP actuator display robust attachment and excellent interfacial adhesion between the electrode and electrolyte layers (Figure S9a,b). Importantly, even after 50,000 cycles of continuous actuation under open-air conditions, the electrode–electrolyte interface remains structurally stable without delamination (Figure S9c,d). The mechanical properties of the MXene–CTF electrode layer compared to those of the pristine MXene and CTF active materials are shown in Table S4.

The all-in-one actuation performance of the MXene-CTF-PP soft actuator was found to be satisfactory and superior for use in practical robotic devices. To gain insight into the advancement of key actuation parameters through the use of distinctive MXene–CTF active material, a comprehensive comparative analysis was undertaken, taking into account the latest developments in this promising field (Figure 5g–i). Figure 5g depicts a record-setting bending strain of 1.1%, accompanied by ultrafast actuation responses of 1.4 s at an ultralow input potential of 0.5 V. This comparative figure clearly illustrates a notable advancement between the MXene-CTF-PP soft actuator and the others reported to date, which have exhibited a bending strain of less than 1.0% even when a voltage exceeding 1.0 V was applied.^44,49a–49n^ A comparison of the phase delay at a common 0.1 Hz excitation frequency reveals that the MXene-CTF-PP electro-ionic soft actuator exhibits the lowest value, which is under 0.15 rad, despite an increase in thickness up to 0.2 mm (Figure 5h).^44,46,47e,49g,49i,49l,49m,50a–50c^ The blocking force, which is of great importance for the precise control of actuator movement against resistive loads under low input energy, was found to be the highest in the MXene-CTF-PP electroactive soft actuator (Figure 5i). The electrochemical capacitive actuation principle, in which only ion movement causes the mechanical bending, presents a significant challenge in obtaining a high blocking force soft actuator. This has led to restricting their use in practical applications where high output force is necessary, such as in biomedical and adaptive soft robotic devices. The MXene–CTF active electrode material enables achieving an unattainable blocking force of 5.80 mN at 2.0 V DC input energy, surpassing the previous reports in the field (Figure 5i).^44,46,47a,47e,47h,49a–49c,49f,49h–49k,49m,49n,50a,51a–51d^ The normalized blocking forces of MXene–CTF–PP soft actuator under various DC applied voltages of (0.5–2.0 V) are summarized in Table S5. This considerable mechanical bending force of the MXene–CTF electrochemical actuator successfully propels objects weighing approximately 25–50 mg over distances of 5–10 cm at an applied voltage of 1.0 V and an excitation frequency of 1.0 Hz (Movie S2 and Figure S10). The synergistic effect of MXene and 4*H*-pyran functionalized CTF active materials made these enhanced performance outcomes possible, which could be further optimized by selecting appropriate CTF precursors with higher electroactive sites and surface areas.

## Conclusions

This study presents a molecularly engineered MXene–CTF hybrid that exhibits unprecedented electrochemical cyclic stability and exceptionally rapid actuation performance in electro-ionic soft actuators. This approach effectively stabilized the surface terminations of Ti_3_C_2_T_*x*_ MXene, prevented oxidation, and significantly enhanced its electrochemical capacitive properties. The 4*H*-pyran unit underwent a robust *H*-bonding electronic interaction with the surface hydroxyl group of MXene at the molecular level, as demonstrated by the *few-nanometer-scale* TEM analysis. This unique combination of two potentially valuable electroactive materials has effectively resolved the most critical challenges associated with supercapacitors and actuators, while exhibiting the necessary electrochemical attributes with notable advancement. The intriguing electrochemical properties of MXene–CTF delivered an eminent energy density of 159.8 Wh kg^–1^ at a power density of 150 W kg^–1^, achieving record values of bending strain (1.1%), blocking force (5.8 mN), and phase delay (≤0.15 rad) under ultralow input potential (0.5 V) with ultradurable and ultrafast actuation speed. This all-in-one MXene–CTF active material has not only significantly advanced the field of electrochemical soft actuators but also demonstrated potential for further regulation of electrochemical properties for device-level applications.

## Methods

### Synthesis of Delaminated Ti_3_C_2_T_*x*_ MXene

The delaminated Ti_3_C_2_T_*x*_ MXene was prepared by following the previously described synthetic method.^46^ In this typical synthetic procedure, a mixture of lithium fluoride (0.077 M, 2 g) and hydrochloric acid (6 M, 20 mL) was utilized as an etchant of aluminum (Al) for Al–Ti_3_AlC_2_ MAX (1 g, 400 rpm, 35 °C, 24 h). Subsequently, the etched solution mixture was shaken using a mechanical shaker (300 rpm, 1 h) prior to neutralization through repeated centrifugations with deionized (DI) water. The multilayer Ti_3_C_3_T_*x*_ was then collected by centrifugation (7000 rpm, 10 min) with 50 mL of DI water. It was then subjected to mechanical shaking (300 rpm, 2 h) to facilitate the complete delamination of each MXene layer, which was finally collected by centrifugation (3500 rpm). The delaminated MXene ink was freeze-dried over 48 h and transferred to an argon-filled glovebox for further utilization.

### Growth of 4*H*-Pyran CTF on the 2D-Layer Surface of Ti_3_C_2_T_*x*_ MXene

The ionothermal growth of 4*H*-pyran CTF was achieved under completely dry and vacuum conditions using anhydrous ZnCl_2_ as both the solvent and the catalyst. To maintain the precursors in an environment free of moisture and oxygen, the glass ampule was charged with Ti_3_C_2_T_*x*_ MXene (20 mg), 4-(dicyanomethylene)-2,6-dimethyl-4*H*-pyran (50 mg, 0.29 mmol), and anhydrous ZnCl_2_ (198 mg, 1.45 mmol) within the glovebox. The charged ampule was then degassed for 12 h at 70 °C prior to sealing by oxidizing flame under vacuum. To initiate the growth of 4*H*-pyran-functionalized CTF, the temperature was increased gradually in a regulated box furnace at a heating rate of 1 °C/min up to 600 °C. The temperature was then maintained at 600 °C for 48 h to allow the completion of the CTF growth. The MXene–CTF, as produced, was then cooled naturally, and the crude material having a black color was collected after washing several times with DI water and finally with acetone. Prior to the additional spectroscopic and electrochemical characterizations, the Soxhlet extraction process was conducted using acetone for a period of 24 h followed by vacuum drying at a temperature of 110 °C for 8 h. The pristine 4*H*-functionalized CTF was synthesized using the same method without adding MXene.

### Fabrication of MXene–CTF Electrochemical Supercapacitor Using Ionic Liquid Electrolyte

The conventional electrode fabrication method was employed, whereby MXene-CTF was thoroughly blended with Super P and poly­(vinylidene fluoride) in a weight ratio of 8:1:1, with a minimal volume of *N*-methyl-2-pyrrolidone. Subsequently, the homogeneous electrode ink was carefully cast onto the top surface of the aluminum current collector and dried under a vacuum at 60 °C for 24 h. The average mass loading of the MXene–CTF active material was calculated to be approximately 4.2 mg, which is equivalent to 2.4 mg cm^–2^ on the aluminum. The resulting MXene-CTF electrodes were assembled in the CR2032 coin-type cells, in which commercial Celgard 3401 (polypropylene) was employed as a separator, and EMIM-BF_4_ ionic liquid was utilized as an active electrolyte. A minimal quantity of electrolyte was maintained for all the electrochemical supercapacitor analyses with an E/C ratio of 5.

### Fabrication of MXene–CTF Electrochemical Soft Actuator Using Ionic Liquid Electrolyte

The MXene-CTF-PP electrochemical soft actuator was fabricated by the previously described method.^44–46^ An electrolyte membrane comprising an ionic liquid (EMIM-BF_4_) in Nafion (120 μm thick) was prepared via solution casting. A homogeneous solution of EMIM-BF_4_ (30 mg mL^–1^) in Nafion (50 mg mL^–1^ in *N*,*N*-dimethylacetamide) was cast on a flat glass Petri dish (5 cm in diameter) and dried in a vacuum oven at 85 °C for 7 h to obtain a free-standing, mechanically flexible electrolyte membrane. Subsequently, a dispersion of MXene–CTF in dimethyl sulfoxide (0.4 mg in 1 mL, sonicated for 20 min) was mixed with an aqueous PEDOT-PSS solution (1%, wt/vol) and stirred for 24 h at 25 °C to ensure homogeneity. The resulting MXene–CTF active electrode ink was then drop-cast onto both sides of the freshly prepared electrolyte membrane, followed by solvent evaporation at 70 °C for 1 h. The effective amount of MXene–CTF in the electrode ink was optimized by assessing the electrochemical ionic capacitance and actuation performance, as detailed in Table S3. The optimal dimensions of the MXene-CTF-PP soft actuator (180 μm thick) were maintained at 20.0 mm and a width of 4.0 mm for electrochemical actuation analysis. A pristine PEDOT-PSS-based soft actuator (PP) was also fabricated using the same procedure and amount of dimethyl sulfoxide to evaluate the precise impact of MXene–CTF on enhancing the electrochemical actuation performance.

### Characterization

The high-resolution SEM and TEM images of the MXene-CTF nanoporous structural surface were obtained using a Hitachi SU8230 scanning electron microscope and a Titan Double Cs-corrected transmission electron microscope (Titan cubed G2 60-300, FEI), respectively. Elemental mapping images were taken at the few-nanometer scales using scanning TEM with a high-angle annular dark-field detector. FTIR analysis was performed by using an FTIR spectrometer (Nicolet iS50) from Thermo Fisher Scientific, and Raman spectra were collected by using a high-resolution Raman spectrometer (LabRAM HR Evolution Visible_NIR, HORIBA). To examine the specific surface area and pore-size distribution, argon physisorption isotherms were recorded at 87 K using a Micromeritics surface area and pore-size analyzer (3Flex). The temperature was maintained constant using a circulator throughout the adsorption and desorption processes. All adsorption–desorption measurements were conducted three times to ensure reproducibility with negligible discrepancies observed in the isotherm points across the experiments. To analyze the electronic interaction between MXene and 4*H*-pyran functionalized CTF, XPS measurements were performed using a Thermo VG Scientific Instrument (K-α) at a base pressure of 3 × 10^–8^ Pa.

The electrochemical CV of MXene–CTF and the supercapacitor were measured by using a workstation, VersaStat3, manufactured by Princeton Applied Research. The conventional three-electrode cell configuration, where Ag/AgCl serves as the reference electrode and platinum foil serves as the counter electrode, was employed to measure the CV responses of the MXene–CTF active electrode.^43^ In a freshly polished glassy carbon electrode, a known amount of MXene–CTF was loaded by using poly­(tetrafluoroethylene) as a binder and *N*-methyl-2-pyrrolidone as a solvent. Both the aqueous (1 M, KOH and H_2_SO_4_) and organic electrolyte (0.5 M, EMIM-BF_4_) solutions were prepared to evaluate the electrochemical charge storage and discharge characteristics of the MXene–CTF active electrode material. The resulting CV data were analyzed to obtain the specific capacitance (*C*_sp_) in F g^–1^ according to eq 1.^43,44^

1 $$C_{\mathrm{sp}}=\frac{A}{2\times \Delta V\times v\times m_{a}}$$

In this equation, *A* represents the area enclosed by the CV curve, Δ*V* denotes the potential window, *v* signifies the scan rate, and *m*_a_ refers to the mass of the active electrode material.

To evaluate the capacitive properties of the MXene–CTF symmetric supercapacitor, the as-fabricated CR2032 coin-type cell was subjected to both electrochemical CV and charge–discharge profile measurements under various scan rates and current densities. Utilizing an ionic liquid electrolyte in the MXene–CTF supercapacitors allows for wider potential windows, from −0.5 to 2.5 V (vs SHE), which is a significant advantage over other aqueous supercapacitor technologies. The specific capacitance of the supercapacitor (*C*_sup_) was calculated from the Galvanostatic charge–discharge response data, which were obtained under the input current densities ranging from 0.1 to 5.0 A g^–1^ by following the procedure outlined in eq 2.^43^

2 $$C_{\sup}=\frac{I\times t_{d}}{\Delta V\times m_{a}}$$

In this equation, *I* represents the input current (mA), *t*_d_ denotes the discharging time (s), Δ*V* signifies the potential window (V), and *m*_a_ refers to the mass of active materials (mg). The *C*_sup_ was thus utilized to calculate the energy density (*E*_d_) and power density (*P*_d_) of the MXene–CTF supercapacitor by eqs 3 and 4.^43^

3 $$E_{d}=\frac{1}{2}\times C_{\sup}\times {\Delta V}^{2}\times \frac{1000}{3600}$$

4 $$P_{d}=\frac{E_{d}\times 3600}{t_{d}}$$

To gain insight into the contact charge resistance and diffusivity of ions in supercapacitors, the frequency was selected within the range of 0.01–500,000 Hz with an amplitude of 50 mV RMS for the EIS measurement.

A laser displacement sensor (Keyence, LK031) was employed to assess electrochemical bending displacement in the MXene-CTF-PP and PP soft actuators under ultralow input potential. The ultralow voltage-driven bending strain difference (ε) between the two opposite electrode layers in the MXene-CTF-PP and PP soft actuators was calculated using eq 5.^44^

5 $$\varepsilon =\frac{2\delta d}{l^{2}+\delta^{2}}$$

In this equation, δ represents the maximum deflection of the soft actuator from its original position, *d* refers to the thickness of the actuator, and *l* signifies the active length of the actuator.

The phase delay (Δφ) for the electrochemical actuation of MXene-CTF-PP and PP soft actuators was calculated by eq 6.^44^

6 Δφ=2πf(Δt)

The symbol Δ*t* represents the time delay, which can be obtained by calculating the time differences between two equivalent points on the input potential signal and the output bending displacement, *f* refers to the input frequency of the actuation signal.

A force sensor (LVS-5GA load cell, Kyowa) with a capacity of 50 mN was utilized to quantify the blocking force of the electroactive soft actuators.^44–46^ Each measurement was repeated five times, and the mean value was calculated.

## Supplementary Material







## Data Availability

The original data that support the findings of this study are available from the authors upon request.

## Notes

TEM images; pore-size distribution; XPS; FTIR; Raman; electrochemical CV; galvanostatic charge–discharge profiles; images of electrochemical soft actuators; cross-sectional SEM images; demonstration images; and Tables S1–S5 (PDF)
Mechanical deflection of the soft actuator under various applied voltages at 1.0 Hz (Movie S1) (MP4)
MXene-CTF-based soft actuator for object propulsion at 1.0 V and 1.0 Hz (Movie S2) (MP4)
