# Supplementary material for: Molecular Engineering of MXene-Covalent-Triazine Framework Interfaces for Electrochemical Actuators
Source: ACS Nano. 2025 Jul 1;19(28):25757–69. doi: 10.1021/acsnano.5c04154 (PMC12365921; doi:10.1021/acsnano.5c04154)
Supplement: Supplementary file 1 [file nn5c04154_si_001.pdf]

Supporting Information

Molecular Engineering of MXene-Covalent-Triazine Framework Interfaces for Electrochemical Actuators

Manmatha Mahato[1+], Sanghee Nam[1+], Geetha Valurouthu[2], Hyunjoon Yoo[1], Mousumi Garai[1], Ji-Seok Kim[1], Woong Oh[1], Jawon Ha[1], Vipin Kumar[1], Chi Won Ahn[3], Yury Gogotsi[2]*, and Il-Kwon Oh[1]**

[1] Dr. M. Mahato, Dr. S. Nam, H. Yoo, Dr. M. Garai, Dr. J.-S. Kim, Dr. W. Oh, J. Ha, Dr. V. Kumar, Prof. I.-K. Oh

National Creative Research Initiative for Functionally Antagonistic Nano-Engineering, Department of Mechanical Engineering, Korea Advanced Institute of Science and Technology (KAIST), 291 Daehak-ro, Yuseong-gu, Daejeon 34141, Republic of Korea

[2] Dr G. Valurouthu and Prof. Y. Gogotsi

Department of Materials Science & Engineering, and A.J. Drexel Nanomaterials Institute, Drexel University, Philadelphia, Pennsylvania 19104, United States

[3] C.W. Ahn

National Nanofab Center (NNFC), Korea Advanced Institute of Science and Technology (KAIST), 291 Daehak-ro, Yuseong-gu, Daejeon 34141, Republic of Korea

[+] These authors contributed equally.

* Correspondence and requests for materials should be addressed to C.W. Ahn, Y. Gogotsi and I.-K. Oh

E-mail: cwahn@nnfc.re.kr, gogotsi@drexel.edu and ikoh@kaist.ac.kr

Keywords: MXene; CTF; functional active materials; supercapacitors; actuators.

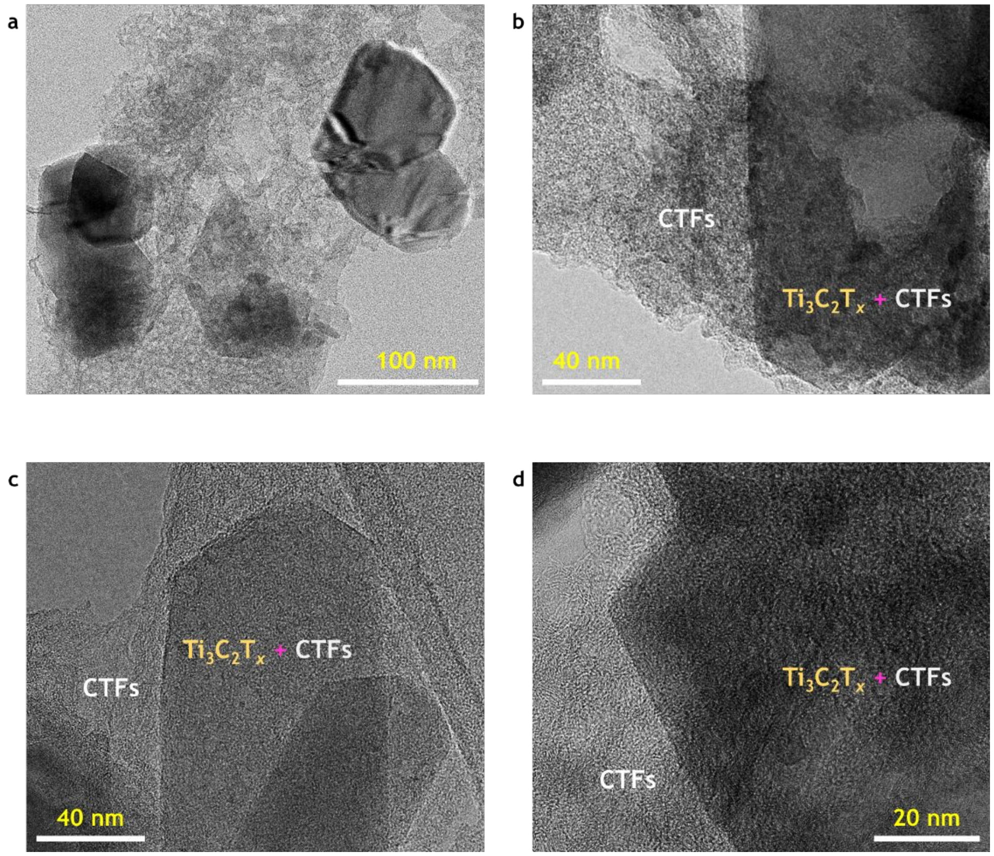

Figure S1. High-resolution TEM images of MXene–CTF at lower magnifications.

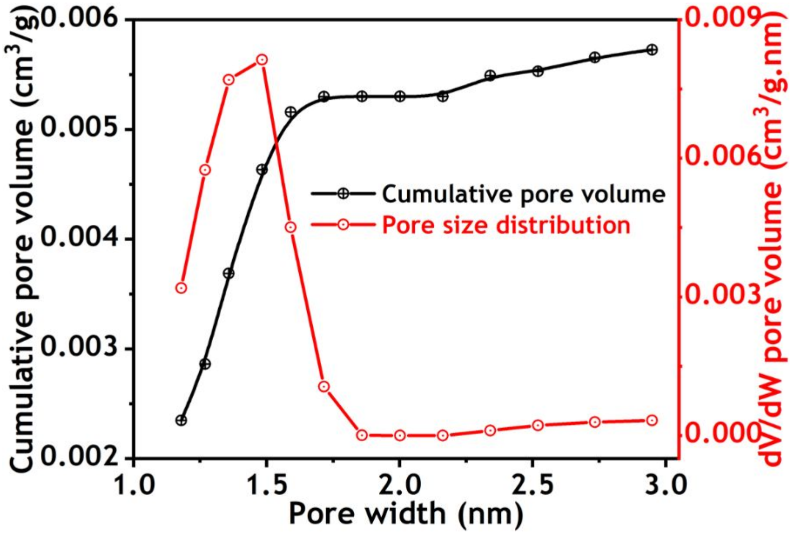

Figure S2. Pore-size distribution of pristine $Ti_3C_2T_x$. Analyzed from Argon-physisorption isotherm.

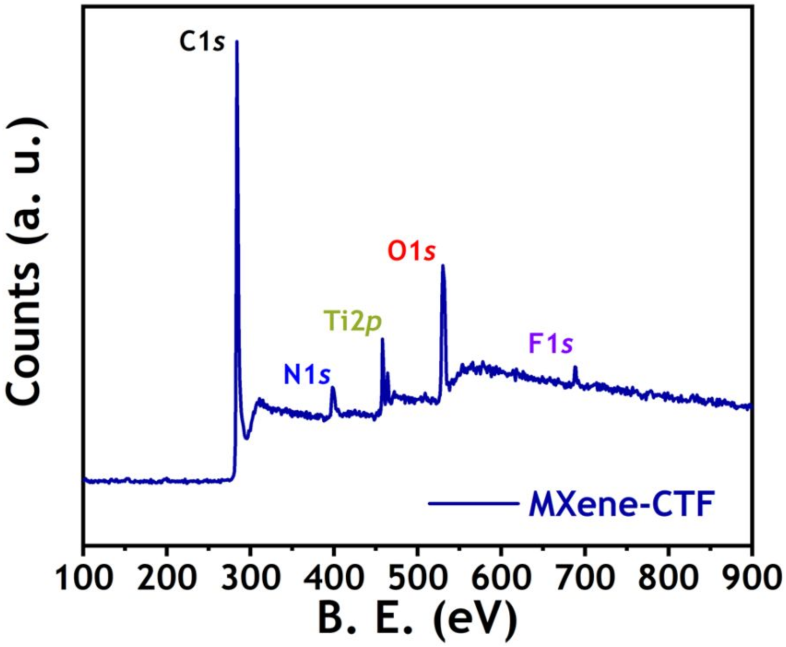

Figure S3. X-ray photoelectron spectroscopic survey analysis of MXene–CTF to confirm the basic building-block elements.

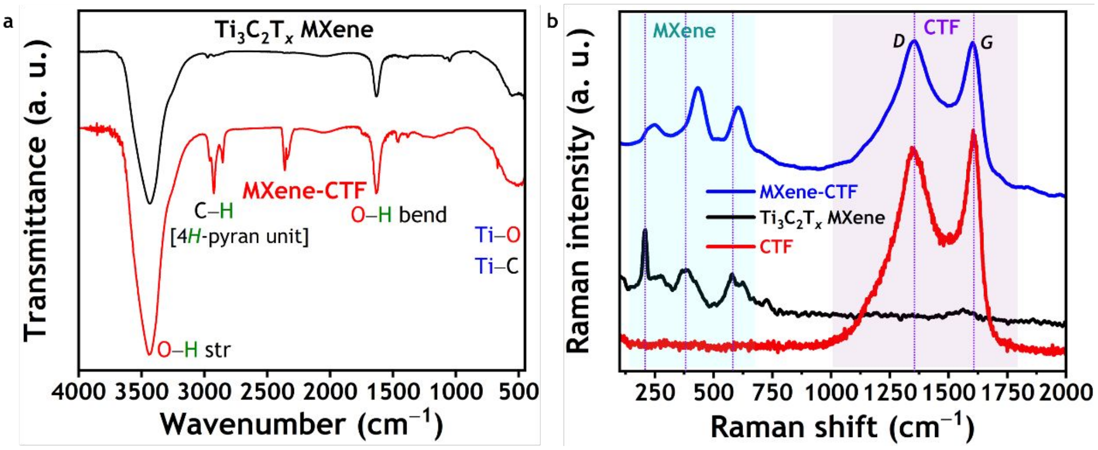

Figure S4. Spectroscopic characterization of MXene–CTF active materials. (**a**) Comparative FTIR spectra of pristine MXene and MXene–CTF. (**b**) Comparative Raman spectra of bare CTF, MXene, and MXene–CTF.

The FTIR spectra confirm the presence of essential functional moieties in both the $Ti_3C_2T_x$ MXene and MXene–CTF materials. However, due to the inherently broad nature of the O–H stretching and bending vibration bands, precise wavenumber shifts related to hydrogen bonding interactions between the oxygen atoms in the 4*H*-pyran groups of CTF and the surface hydroxyl groups of MXene could not be distinctly resolved (Figure S4a).

The Raman spectra (Figure S4b) of the MXene–CTF exhibit characteristic peaks originating from both $Ti_3C_2T_x$ MXene [35] and the 4*H*-pyran functionalized CTF [43]. Comparative analysis with pristine CTF and MXene shows that the Raman bands associated with condensed and disordered triazine frameworks (*G* and *D* bands) remain unaltered. However, a slight shift towards higher frequencies was observed for characteristics MXene Raman bands. These results indicate that nitrogen atoms within the triazine frameworks of CTF are not significantly involved in electronic interactions. Instead, oxygen atoms in the 4*H*-pyran units interact prominently through hydrogen bonding with the hydroxyl groups on the MXene surface. These observations strongly support our detailed XPS analysis of interfacial interactions (Figure 3c, d), confirming that electronic interactions at the interface occur exclusively between the 4*H*-pyran oxygen atoms in CTF and the hydroxyl groups of MXene, while nitrogen atoms remain uninvolved.

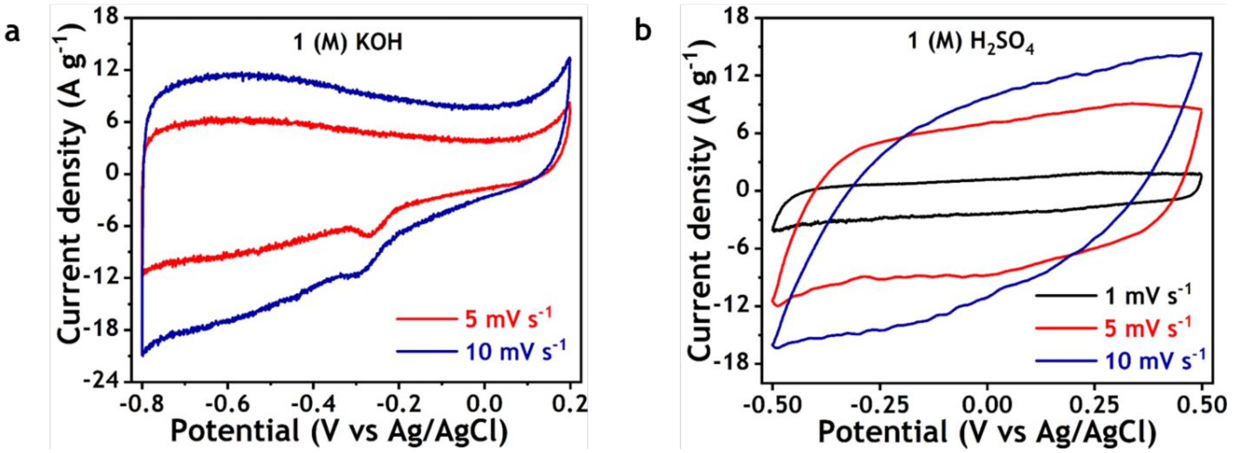

Figure S5. Electrochemical cyclic voltammetry (CV) response patterns of MXene–CTF in aqueous electrolytes: (a) 1.0 M KOH and (b) 1.0 M H_2SO_4.

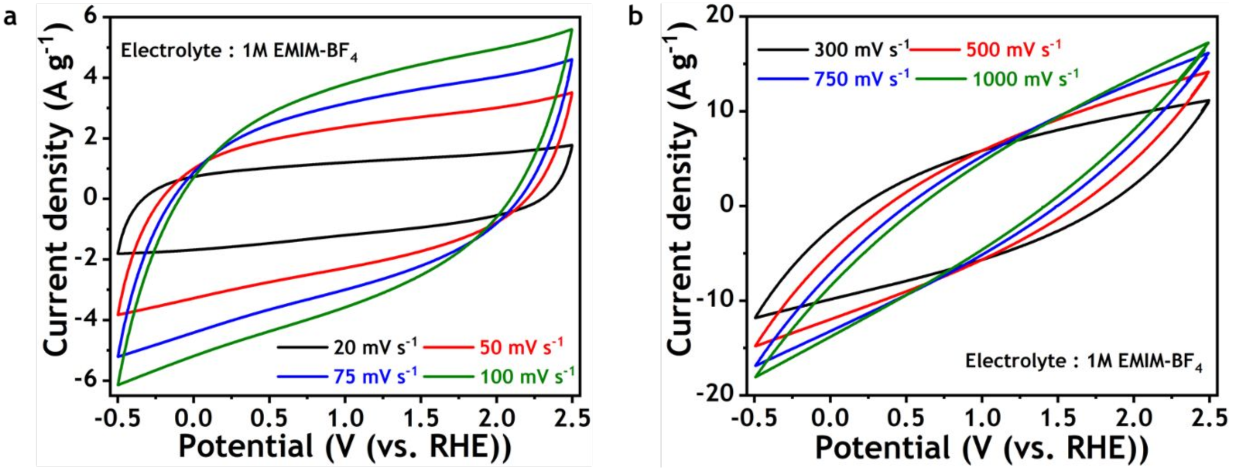

Figure S6. Electrochemical CV response patterns of MXene–CTF supercapacitor in a non-aqueous EMIM-BF_4 electrolyte at increasing scan rates: (a) 20–100 mV s^{-1} and (b) 300–1000 mV s^{-1}.

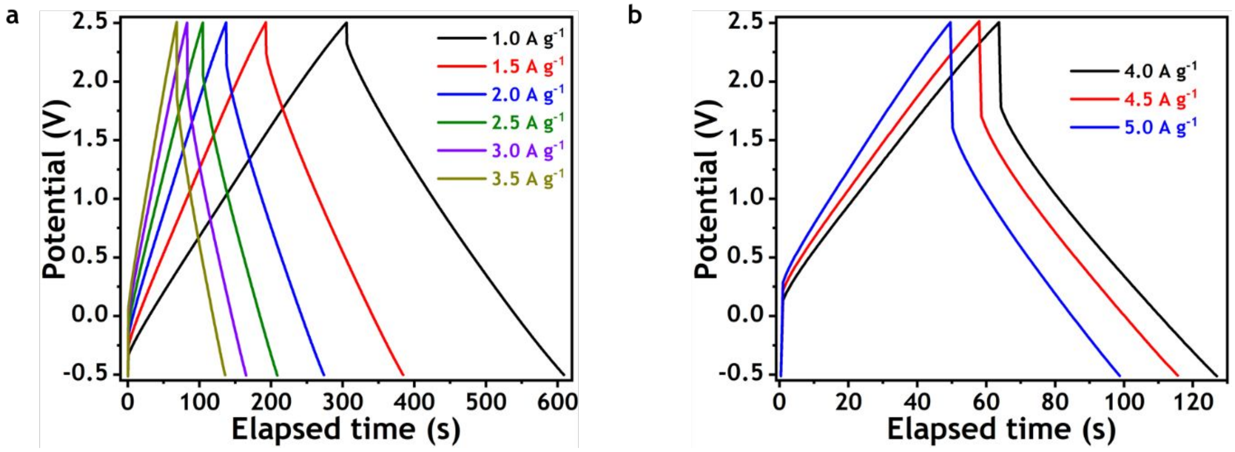

Figure S7. Galvanostatic charge-discharge profiles of MXene–CTF supercapacitor in a non-aqueous EMIM-BF_4 electrolyte at increasing input current densities: (a) 1.0–3.5 A g^{-1} and (b) 4.0–5.0 A g^{-1}.

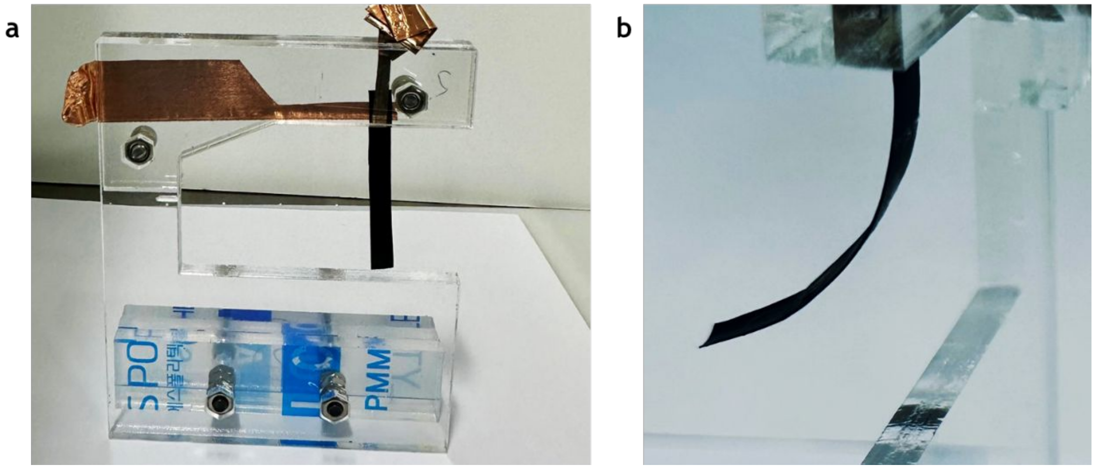

Figure S8. Optical images of the MXene–CTF based electrochemical soft actuator. (**a**) Custom-built actuation setup with the actuator installed. (**b**) Actuator mechanical deflection under applied DC voltage of +0.5 V.

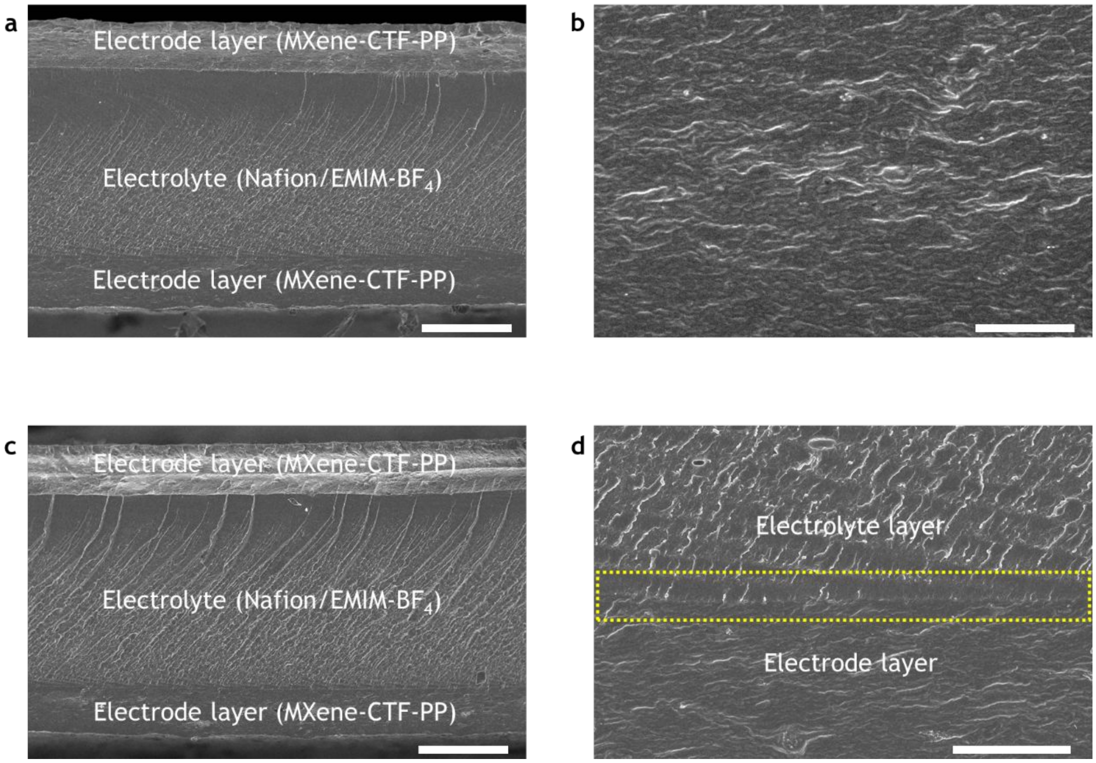

Figure S9. Cross-sectional SEM images of MXene–CTF electrochemical actuator. (**a**) Cross-sectional view of the as-prepared actuator (scale bar: 50 μm). (**b**) Magnified view of the cross-sectional electrode layer (scale bar: 5 μm). (**c**) Cross-sectional image of the actuator after 50,000 cycles of continuous excitation at 0.5 V and 1.0 Hz (scale bar: 50 μm). (**d**) Magnified view of the stable interface between the electrode and electrode layers after prolonged cycling (scale bar: 10 μm).

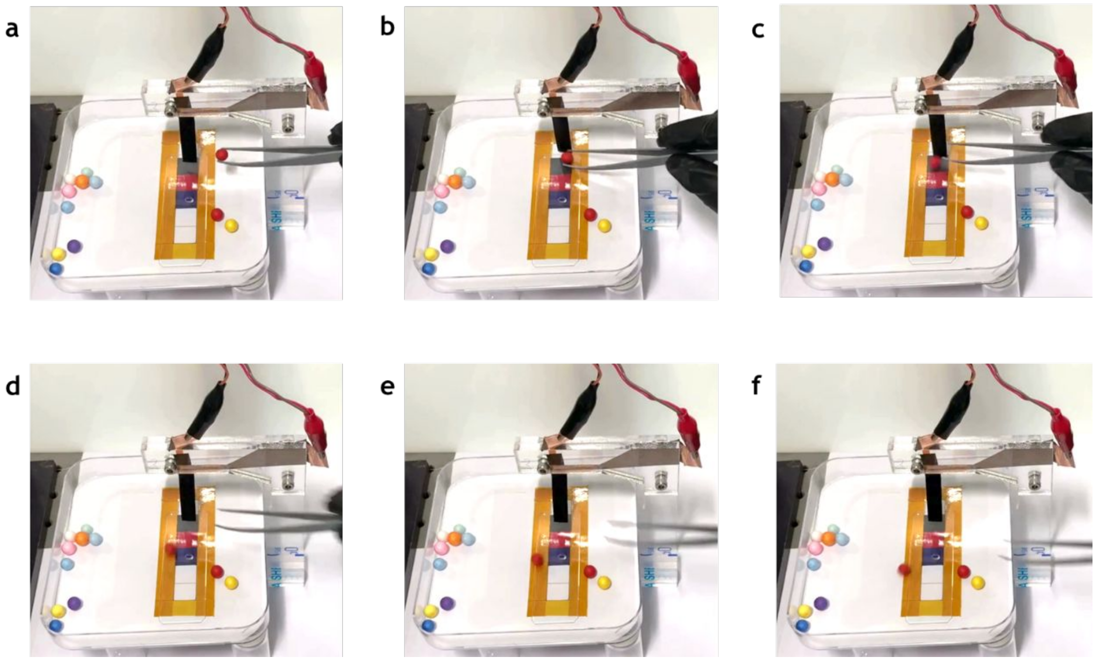

Figure S10. Demonstration of the electro-ionic MXene–CTF based soft actuator propelling ball at an applied voltage of 1.0 V and an excitation frequency of 1.0 Hz. (**a–c**) Positioning of the ball within the actuation area of the soft actuator. (**d–f**) Sequential images showing the ball being propelled by the actuator.

Table S1. Ionic specific capacitance of bare CTF, MXene and MXene–CTF in EMIM-BF_4/acetonitrile electrolyte solution.

Active material	Ionic specific capacitance (F g^{-1}), @ 10 mV s^{-1}
CTF	166
MXene	271
**MXene–CTF**	**626**

Table S2. Comparison of ionic specific capacitances for high-performance active electrode materials in non-aqueous ionic-liquid electrolyte solution at a scan ate of 10 mV s^{-1}.

SN	Active electrode material	Ionic specific capacitance (F g^{-1})	Ref
1	Th-SNG	503	[47a]
2	HLrGOP	165	[47b]
3	PZMOF-2	605	[47c]
4	3D G–CNT–Ni	434	[47d]
5	NG	248	[47e]
6	TP6	437	44
7	DCB-TF-600	295	45
8	DDP600	271	43
9	LSG	276	6
10	PICA	280	[47f]
11	BS-COF-C900	389	[47g]
12	3D GCN-NG	426	[47h]
13	MXene/CNT	150	[25]
14	MPA10	455	[26]
**13**	**MXene-CTF**	**626**	**This work**

Table S3. Specific capacitance of MXene–CTF supercapacitor at varying current based on electrochemical discharge profiles.

Current density (A g^{-1})	Discharge time (s)	Specific capacitance (F g^{-1})
0.1	3834.0	127.80
0.2	1786.5	119.10
0.3	1142.0	114.20
0.4	832.0	110.93
0.5	647.5	107.92
0.75	418.0	104.50
**1.0**	**303.5**	**101.17**
1.5	191.5	95.75
2.0	136.5	91.00
2.5	104.0	86.67
3.0	82.5	82.50
3.5	67.5	78.75
4.0	56.5	75.33
4.5	48.0	72.00

5.0	41.0	68.33

Table S4. Mechanical properties for MXene–CTF-PP, MXene-PP, and CTF-PP electrode layers.

Electrode layer	Young's modulus (MPa)	Tensile strength (MPa)	Elongation at break, %
CTF–PP	53.47	6.52	33.28
MXene–PP	46.71	5.84	38.66
MXene–CTF–PP	65.92	8.45	34.80

Table S5. Blocking forces of MXene–CTF based soft actuator under DC applied voltages.

Applied voltage (V)	Average blocking force (mN)	Standard error (mN)	Normalized blocking force
0.5	2.26	0.005	16.178
1.0	3.83	0.008	27.417
1.5	4.47	0.011	31.998
2.0	5.80	0.015	41.519

The actuator exhibited substantial blocking forces of 2.26, 3.83, 4.47, and 5.80 mN at applied voltages of 0.5, 1.0, 1.5, and 2.0 V, respectively, corresponding to approximately 16, 27, 31, and 41 times its own weight.
